# Correction: Spatial modeling and ecological suitability of Ebola virus disease in Africa

**DOI:** 10.1371/journal.pone.0329763

**Published:** 2025-08-04

**Authors:** Lombo Baluma Didier, Lukusa Lumu Jude, Esuka Igabuchia Franck, HaoNing Wang, Xiao-Long Wang

The first author, Lombo Baluma Didier, is incorrectly noted as the corresponding author. The correct corresponding author is Xiao-Long Wang. Xiao-Long Wang’s email address is: nefuwxl@hotmail.com
