# Supplementary material for: Spatial modeling and ecological suitability of Ebola virus disease in Africa
Source: PLoS One. 2024 Oct 22;19(10):e0311936. doi: 10.1371/journal.pone.0311936 (PMC11495548; doi:10.1371/journal.pone.0311936)
Supplement: Attachment — Submitted filename: Response letter to the Editors and Reviewers.docx [file pone.0311936.s001.docx]

**Response letter to the Editors and Reviewers’ comments**

**Submission ID:** [PONE-D-22-15162]

Lombo Baluma Didier^12^, Lukusa Lumu Jude^3^, Esuka Igabuchia Franck^4^, HaoNing Wang^5,6^, Xiao-Long Wang^12^*

Dear Editor and Reviewers:

We are very grateful for the reviews provided by the editors and each of the external reviewers of this manuscript. The invaluable comments helped us improve the quality of this manuscript greatly. As detailed below, the reviewers’ concerns have been carefully and thoroughly addressed. Our hope is that editors and reviewers ﬁnd the responses convincing and substantial.

Yours sincerely

XiaoLong Wang PhD.

**Journal requirements**

1.Please ensure that your manuscript meets PLOS ONE's style requirements, including those for file naming. The PLOS ONE style templates can be found at
2.<https://journals.plos.org/plosone/s/file?id=wjVg/PLOSOne_formatting_sample_main_body.pdf> and
3.<https://journals.plos.org/plosone/s/file?id=ba62/PLOSOne_formatting_sample_title_authors_affiliations.pdf>

***Response:*** *Thanks for your valuables comments. We downloaded PLOS ONE formatting sample pdf document and followed the journal styles and named the files correctly.*

4.Please ensure that you refer to Figure 1-3 and 5 in your text as, if accepted, production will need this reference to link the reader to the figure.

***Response:*** *Thanks. We have arranged all our figures in such a way that they are quoted in order.*

5.Please include your tables as part of your main manuscript and remove the individual files. Please note that supplementary tables (should remain/ be uploaded) as separate ""supporting information"" files

***Response****: Thanks for your suggestions. We have included all our tables in the main text, adding some tables as suggested by reviewers and in order.*

6.We note you have included a table to which you do not refer in the text of your manuscript. Please ensure that you refer to Table 1 in your text; if accepted, production will need this reference to link the reader to the Table.

***Response:*** *Thank you it done.*

**Reviewer #1:**

**Introduction**

1.The introduction is quite wordy, I suggest removing some text, for example, the paragraphs…” The first outbreaks of EVD occurred in isolated villages in central Africa [citation needed] …….”. The next two paragraphs following this are also redundant.

*Response: We have enlarged the paragraph starting with the first outbreaks of EVD occurred in isolated villages in central Africa and the two paragraphs that follow this paragraph.*

2.Please try to focus on why this study is important and how it is different from other studies conducted.

***Response:*** *This study is essential as the spatial modeling techniques have been applied to predict EVD outbreaks, which has shown potential in another zoonotic disease. Epidemiological studies applying Species Distribution Models, such as MaxEnt, provide a robust framework for identifying the drivers of disease spread from environmental and socio-demographic factors [20,21].*

3.Introduction could benefit from a review on modeling other zoonotic diseases that applied the same techniques as this study.

***Response:*** *Several such studies related to the present one are: Mapping the zoonotic niche of Ebola virus disease in Africa [17], Assessing the zoonotic risk of Ebola virus disease in West Africa using a spatial modeling framework [18], and Mapping of Ebola virus spillover: Suitability and seasonal variability at the landscape scale [19]. All these three papers used multicriteria decision analysis (MCDA) methods integrated in GIS; the last two papers are limited to only a few African countries. This paper, however uses a different methodology and has constructed a model which is extendable by including additional variables.*

4.Please provide some information on the available modeling approaches or SDMs such as the one this study has used (i.e., Maxent) for epidemiological studies.

***Response:*** *Recent innovations in the applications of SDM have illuminated the dispersal of several zoonotic infections, and extrapolation to Ebola is feasible. These modeling techniques have been used for studies that are somewhat analogous, particularly regarding Lyme disease and Hantavirus, concerning the vector-host-environment scenario [15,16]. The models, such as MaxEnt, define potential risk areas and are especially useful when planning targeted public health initiatives. To compare the predictive power of the MaxEnt model with other epidemiologic models and validate their effectiveness in forecasting disease.*

5.Please outline the objectives of the study clearly at the end of the introduction.

*Response: We put the objectives of this study in this end of introduction. This work intends to (1) map distribution patterns of known EVD, (2) make predictions on possible at-risk areas, and (3) identify dominant environmental variables associated with EVD outbreaks so both national and international policymakers can target appropriate prevention efforts and implement effective surveillance strategies.*

**Materials and Methods**

6.I suggest restructuring the methodology section into the following subsection:
(1) Occurrence points
(2) Environmental variables
(3) Model building (providing details on model parameterization is also necessary, e.g., information of the background points of the Maxent model)
(4) Model evaluation

***Response:*** *We have restructured the methodological part as requested by reviewer 1 and for point 3 we have added this: We adopted the Maximum Entropy (MaxEnt) modeling approach because it is one of the best ways to implement ecological niche models, even when dealing with small amounts of data [26, 28]. The MaxEnt model run was allowed a maximum of 5000 iterations during its run, with the convergence threshold set at 0.00001, and up to 5000 background points were run. A 70/30 splitting approach was used when partitioning between training and test datasets for testing the predictive performance of the model. Presence pseudo-absence locations were randomly located throughout the model study area to represent potential background points, enhancing model discrimination capability between suitable and unsuitable habitats for EVD [26, 28].*

7.“Area Under the Receiver Operating Curve (AUC)”; this metric alone, sometimes, is not sufficient as the only evaluation metric. Please justify why only AUC was used. In this section, it's important to clarify what threshold was used to delineate the suitability and unsuitability areas. These questions should be answered in the methodology section.

***Response:*** *The overall performance evaluation was based on the selected primary metric: the Area Under the Receiver Operating Characteristic Curve (AUC). AUC is applicable especially for the measurement of the discriminatory performance of the model, i.e., how well the model can distinguish areas with the presence or absence of EVD for all probability cut-offs. Our model had an average AUC of 0.987; thus, it performed very well [29]. Although robust, the AUC is sensitive to the choice of threshold for classifying suitable and unsuitable conditions. We also used the jackknife test to determine which variables are least/most important, hence for reduced area tests. We used response curve analyses to determine the sensitivity of predictions to the threshold chosen [30]. We identified the suitability and unsuitability threshold using the criterion that gives the most significant sum of sensitivity and specificity. This threshold optimization is essential to ensure that the model's prediction closely resembles the observed distribution patterns of EVD, hence reliable maps for public health planning and resource allocations [30,31].*

**Results:**

8.Results are also required to be structured similar to the methods i.e., 1,2,3,4, and each subsection should be reported clearly.

***Response:*** *The result was arranged according to this suggestion and each session was very well clarified.*

**Discussion**

9.Discussion requires two subsections in which the the limitations of the modeling technique should be discussed. In addition to this, a small section highlighting the benefits of the applied modeling techniques, i.e., combing geospatial techniques with Maxent and identifying relevant factor preferences in establishing priority zones for management actions is necessary. For these suggestions I recommend these references:
10.<https://www.mdpi.com/2071-1050/15/18/13669>
11.<https://doi.org/10.1016/j.ecoinf.2022.101930>
12.<https://doi.org/10.1007/s10661-024-12438-z>

***Response:*** *We have inserted two sessions into the discussion, the first is 4.1. Benefits of the Applied Maxent Modeling Techniques and the second Limitations of the MaxEnt Modeling Technique. We are then adapting our discusion, taking into account the links to the papers sent to us by the reviwiers.*

**Reviewer #2:**

1.Supplement descriptions of data quality control and validation steps

***Response:*** *In order to provide further clarity regarding the quality control of our data and the different steps of validation, we have added an additional description in Line ?-?.*

2.The paper uses SDM Toolbox v1.1c to handle spatial autocorrelation, setting a minimum distance of 10 km. However, there is a lack of detailed explanation regarding the methods and criteria for this spatial autocorrelation treatment. It is suggested to elaborate on the rationale for choosing 10 km as the minimum distance and how this setting affects the stability and accuracy of the results.

***Response:*** *We've added two sub-sessions to the occurrence point session to explain why we've chosen 10km as the minimum for the use of SDM Toolbox v1.1c to handle spatial autocorrelation. Please refer to Line ?-?.*

3.Provide specific results of PCA and VIF testing.

***Response:*** *Two additional tables have been incorporated into the third section of the methodology. One table presents the results of the principal component analysis (PCA) (Table 1), while another displays the results of the variance inflation factor (VIF) analysis (Table 2).*

4.Use more evaluation metrics to comprehensively assess the model's performance.

*Response: Thank you for your valuable suggestions. Based on your feedback, we have included additional evaluation metrics to comprehensively assess the model's performance. Below are the specific improvements we made and the results obtained:*

*Improvements*

5.Although the results show significant impacts of population density and rainfall patterns on EVD, the practical application of these results can be further explored. It should be discussed how to translate these findings into practical applications, specifically how public health measures should be formulated and implemented based on these results. For example, how these findings can be specifically applied to public health interventions and policy-making.

***Response:*** *The results were discussed in detail in Line, demonstrating how our findings could be applied to public health by assisting decision-makers in implementing effective and responsible measures to manage the population in order to prevent the outbreak of the Ebola virus in the future.*

*For your convenience, we paste it below:*
